# Time-to-Positivity for Candida in Bloodstream Infections: Prognostic Implications for Mortality

**DOI:** 10.7759/cureus.66364

**Published:** 2024-08-07

**Authors:** Lavanya Balaji, Harish Manoharan, Neelusree Prabhakaran, Nandhagopal Manivannan

**Affiliations:** 1 Department of Microbiology, Saveetha Medical College and Hospitals, Saveetha Institute of Medical and Technical Sciences, Saveetha University, Chennai, IND

**Keywords:** fungemia, crbsi, candidemia, candida associated mortality, time to positivity

## Abstract

Background

*Candida*-associated catheter-related bloodstream infections (CRBSIs) present a significant challenge in clinical settings, particularly among patients with central venous catheters (CVCs). Time-to-positivity (TTP) of blood cultures, an indicator of fungal load, may provide insights into infection prognosis and severity. This study evaluates the role of TTP in *Candida*-associated bloodstream infections and its impact on patient outcomes.

Materials and methods

This cross-sectional observational study, conducted from July 2023 to June 2024 at Saveetha Medical College, involved collecting blood cultures from intensive care unit (ICU) patients with suspected candidemia. Blood cultures were processed using the BacT/ALERT 3D system (bioMérieux, Marcy l'Étoile, France), with TTP recorded for each *Candida*-positive culture. Species identification was performed using MALDI-TOF MS (Bruker Daltonics, Germany). Species-specific 30-day mortality was analyzed to assess the impact of TTP on survival.

Results

Of 7447 blood cultures from ICU patients, 2349 were positive, with a 2.42% prevalence of *Candida sp*. Among 57 candidemia patients, the median TTP for deceased patients was 24 hours, compared to 25 hours for survivors (p=0.001). *C. auris* exhibited the highest mortality rate (56.25%) with a median TTP of 16.5 hours, whereas *C. albicans* had no associated mortality and a median TTP of 28.5 hours. Shorter TTP was consistently associated with higher mortality across *Candida species*.

Conclusion

This study highlights the prognostic value of TTP in *Candida*-associated bloodstream infections, with shorter TTP correlating with higher mortality. The findings underscore the need for rapid diagnosis and aggressive treatment, particularly for high-risk species like *C. auris* and *C. glabrata*. Further research is needed to refine the clinical application of TTP and develop targeted treatment strategies.

## Introduction

*Candida*-associated catheter-related bloodstream infections (CRBSIs) are a significant concern, especially in patients with central venous catheters (CVCs) [1]. These infections occur when *Candida* species, a type of yeast, colonise the catheter and enter the bloodstream, causing systemic infection. Various factors, such as frequent use of antibiotics and immunosuppressive agents, retained central venous catheters and other invasive devices, underlying chronic illnesses like malignancy, and long stays in the intensive care unit (ICU), increase the risk of *Candida* infection in patients [2]. Time-to-positivity (TTP) for *Candida* species in blood cultures is a parameter that may help predict the prognosis of bloodstream infection (BSI) with *Candida* [3]. TTP refers to the time it takes for blood cultures to become positive after incubating. In the context of CRBSIs, comparing the TTP of cultures drawn from a central venous catheter (CVC) and a peripheral vein can help determine if the catheter is the source of the infection [4]. A higher organism load might be expected in the blood of patients with a more serious disease, resulting in a shorter TTP in blood cultures. Indeed, the majority of data support an association of shorter TTP with higher mortality in BSI, including candidemia [5]. It is important to consider that time to positivity (TTP) can be challenging to interpret and should be analyzed carefully. From a microbiological perspective, TTP indirectly provides information about the biomass, which is influenced by the level of candidemia and the microbial growth rate. A shorter TTP indicates a higher level of microorganisms in the blood or a faster growth rate. TTP is affected by factors often overlooked in routine practice, such as blood volume in the bottles, culture conditions, transportation time, concurrent antimicrobial therapy, and the type of blood sample [6]. Limited research has addressed these factors, but a study by Cobos-Trigueros et al. demonstrated the combined impact of logistics (transportation, laboratory hours), culture media, atmosphere, and microorganism species on the TTP of *Candida glabrata* in blood cultures. Furthermore, the study revealed differences between centers in bottle loading practices, with some loading round the clock and others only during laboratory hours [7]. Diagnostics aimed at identifying the potential source of infection - such as radiographic exams and urine analysis - can be completed within a few hours. However, distinguishing candidemia from non-candidemia infection still takes a considerable amount of time, as reliable alternatives to conventional blood culture incubation are not yet widely used in clinical practice. Biomarkers for ruling out candidemia either lack sensitivity or have practical limitations [8]. Understanding the distribution of blood culture time to positivity (TTP) is clinically advantageous when re-evaluating patients with symptoms consistent with infection. A low likelihood of candidemia may influence the differential diagnosis and subsequent diagnostic and therapeutic decisions if blood cultures remain negative after 48 hours. Therefore, evaluating TTP is a valuable method for diagnosing *Candida*-associated CRBSIs. By comparing the TTP of blood cultures from a CVC and a peripheral vein, clinicians can determine if the catheter is the likely source of infection. This method of clinical assessment and other diagnostic tools can guide appropriate management and treatment strategies. Though an increasing number of TTP-associated articles were published in the past decade, there is still a notable knowledge gap on the outcome prediction of TTP. Despite many articles had analyzed the correlation of TTP and patient outcome, the application of TTP requires further study. Therefore, this study aims to evaluate the time to positivity (TTP) in different* Candida* species causing bloodstream infections and to examine how TTP influences the outcomes of *Candida*-associated bloodstream infections.

## Materials and methods

Study design and setting

This cross-sectional observational study was conducted over one year, from July 2023 to June 2024, at the Central Laboratory, Department of Microbiology, Saveetha Medical College, and Hospital. Blood culture sets were collected from patients suspected of catheter-related bloodstream infections in the intensive care unit (ICU) and processed in the Department of Microbiology after obtaining ethical clearance referenced as 259/07/2023/PG/SRB/SMCH. Before participating in the study, all patients or their legal guardians provided informed consent. The signed consent forms were gathered and securely stored separately from the study data to ensure privacy. All patient data was handled with strict confidentiality. Personal identifiers were either removed or anonymized before data analysis to prevent the identification of individual patients from the data set. Access to the data was restricted to researchers directly involved in the study. Data was shared with external parties only in aggregated or de-identified format to maintain confidentiality.

Blood culture collection and processing

At least two sets of blood samples were collected from different sites, with up to 10 mL for adults, up to 5 mL for pediatric patients, and up to 1 mL for neonates before the administration of any antimicrobials. These samples were inoculated into both aerobic and anaerobic culture bottles. These were incubated using the BacT/Alert automated blood culture system (bioMerieux, Marcy l'Etoile, France). By taking blood from different sites and comparing the results, clinicians can distinguish between a true bloodstream infection and potential contamination from skin flora or other sources. If *Candida* grows from both sets of cultures, it is more likely to be a true infection rather than a contaminant [9]. Larger sample volumes enhance detection sensitivity by optimizing pathogen recovery in cases of low microbial burden and overcoming dilution effects [10]. Time to positivity (TTP) for each patient was determined using the hospital's automated blood culture instrument. Microbial growth was monitored by detecting CO_2_ production with a fluorescent-based detector, and an automated alert signal indicated a positive blood culture.

Time to positivity (TTP) recording

TTP (Time to Positivity) was defined as the time between the start of incubation and the time that the automated alert signal indicating growth in the culture bottle sounded [11]. For each episode of candidemia, the shortest TTP of the first blood culture to become positive with *Candida* spp. during one hospital stay was recorded. Each patient was included in the study only once. Differential Time to Positivity (DTP) was calculated as the difference in TTP between blood cultures drawn from the CVC and those drawn peripherally. A significant DTP suggested that the catheter was the likely source of the infection [12].

*Candida* species isolation and identification

A total of 57 clinical strains of *Candida* species isolated from blood cultures and flagged positive by the BacT/ALERT 3D system (bioMérieux, Marcy l'Étoile, France), were collected from candidemia patients in the ICU. Species identification was confirmed using matrix-assisted laser desorption/ionization time-of-flight mass spectrometry (MALDI-TOF MS) with the Biotyper database v3.1 (Bruker Daltonics, Germany).

Statistical analysis

The sample size was calculated using SPSS software version 21 for Windows (IBM Corp., Armonk, NY, USA). All data was compiled in Microsoft Excel, and the analysis was conducted using SPSS software version 21 for Windows (IBM Corp., Armonk, NY, USA). Descriptive statistics, such as frequencies and percentages, were used to analyze the age and gender distribution of patients diagnosed with *Candida*-associated bloodstream infections. The median and interquartile range (IQR) for the patients' age were calculated to indicate central tendency and dispersion. The Mann-Whitney U test was employed to compare the time to positivity of blood cultures between deceased patients and survivors. Kaplan-Meier survival curves were employed to estimate the cumulative survival probabilities of all *Candida* species based on their time to positivity. Log-rank tests were utilized to compare survival curves across different *Candida *species. In these curves, the horizontal axis represents TTP in hours, ranging from 0 to 100 hours, while the vertical axis illustrates the overall survival probability. Data points marked as "censored" indicate subjects who have died and are not included in the denominator at those time points. Logistic regression analysis was performed to investigate the influence of TTP on 30-day mortality. A p-value of < 0.05 was considered to represent statistical significance.

## Results

From July 2023 to June 2024, 7,447 blood cultures were received from ICU patients, of which 2,349 cultures were flagged positive. The isolation rate of *Candida* species isolated from blood cultures during the one-year period is 2.42%.

Age-wise distribution in candidemia patients in a tertiary hospital

Out of 57 patients diagnosed with candida-associated catheter-related bloodstream infections, the distribution of age groups is as follows: 40.4% (n=23) were aged 40-60 years, 26.3% (n=15) were in the 20-40 years age group, 22.8% (n=13) were over 60 years, and 10.5% (n=6) were less than one year old. The descriptive statistics of age in candidemia patients are presented in Table 1.

**Table 1:** Descriptive statistics of age in candidemia patients.

Parameter	Mean	SD	Median	IQR	Minimum	Maximum
Age (in years)	44.43	20.78	48	29.50	0 (newborn)	78

Gender-wise distribution in candidemia patients in a tertiary hospital

Out of the 57 patients with Candida-associated bloodstream infection, 66.7% (n=38) were males and 33.3% (n=19) were females.

Frequency distribution of risk factors in candidemia patients in ICU

The risk factors that predominantly contributed to candidemia include prolonged broad-spectrum antibiotic usage > 15 days (33%), presence of central venous catheter > 5 days (21%), type 2 diabetes mellitus (10%), sepsis (9%), renal failure (9%), and others.

The demographic characteristics of the candidemia patients are presented in Table 2 and are as follows:

**Table 2:** Demographic characteristics of the candidemia patients The data is presented in N (%)

	Frequency (n)	Percent (%)
Age Distribution (in years)		
<1 year	6	10.5
20 -40 years	15	26.3
40 -60 years	23	40.4
>60 years	13	22.8
Total	57	100.0
Gender Distribution		
Male	38	66.7
Female	19	33.3
Total	57	100.0
Risk Factor Distribution (n=57)		
Renal failure	14	24.5
Cardiovascular impairment	3	5.2
Diabetes mellitus	15	26.3
Preterm	4	7.0
Sepsis	14	24.5
Mechanical Ventilation	2	3.5
Immunosuppression	1	1.7
Total parenteral nutrition	10	17.5
Central Venous Catheter > 5 days	31	54.3
Patient on chemotherapy	6	10.5
Prolonged Broad-spectrum antibiotics	50	87.7

*Candida* species identification

*Candida* species was identified by Matrix-Assisted Laser Desorption/Ionization Time-Of-Flight Mass Spectrometry (MALDI-TOF MS). *Candida albicans* was identified in eight cases, accounting for 14% of the isolates. *Candida tropicalis* was the second most common species, identified in 15 cases, representing 26.3% of the isolates. *Candida glabrata *and *Candida parapsilosis* were each identified in seven cases, both comprising 12.3% of the total isolates. The most frequently isolated species was *Candida auris*, found in 16 cases, making up 28% of the isolates. Less common species included *Candida guilliermondii* and* Candida orthopsilosis*, each identified in two cases, accounting for 3.5% of the isolates. Table 3 shows the distribution of organisms based on MALDI-TOF identification.

**Table 3:** Distribution of identification of organism based on MALDI-TOF MS MALDI-TOF MS: matrix-assisted laser desorption/ionisation time-of-flight mass spectrometry The data is presented in N (%)

Organism identified	No of the organisms identified N (%)
Candida albicans	8 (14%)
Candida tropicalis	15 (26.3%)
Candida glabrata	7 (12.3%)
Candida parapsilosis	7 (12.3%)
Candida auris	16 (28%)
Candida guilliermondii	2 (3.5%)
Candida orthopsilosis	2 (3.5%)

Correlation of outcome with time to positivity in candidemia patients

The Mann-Whitney U test was utilized to analyze the differences between candidemia patients who survived and those who did not across with time to positivity of cultures. The results reveal significant differences in these parameters between the two groups, with p-values <0.05, indicating strong statistical significance, as shown in Table 4. The median time to positivity for blood cultures in deceased patients was 24 hours, compared to 35 hours for survivors. The mean rank was 24.42 hours for deceased patients and 31.29 for survivors, with a p-value of 0.001.

**Table 4:** Comparison of time to positivity with outcomes in Candidemia patients *The P-value <0.05 is considered statistically significant. The p-value was calculated using the Mann-Whitney U test. The p-value is 0.01 The data is presented in N (%)

Mann-Whitney U test	Time to Positivity (in hours)	
	Death (N = 19)	Survivor (N = 38)
Median	24	35
Interquartile Range	25	34.5
Mean rank	24.42	31.29
U value	274.00	
P-value	0.001*	

Correlation between time to positivity and 30-day mortality in candidemia patients

**Figure 1:**
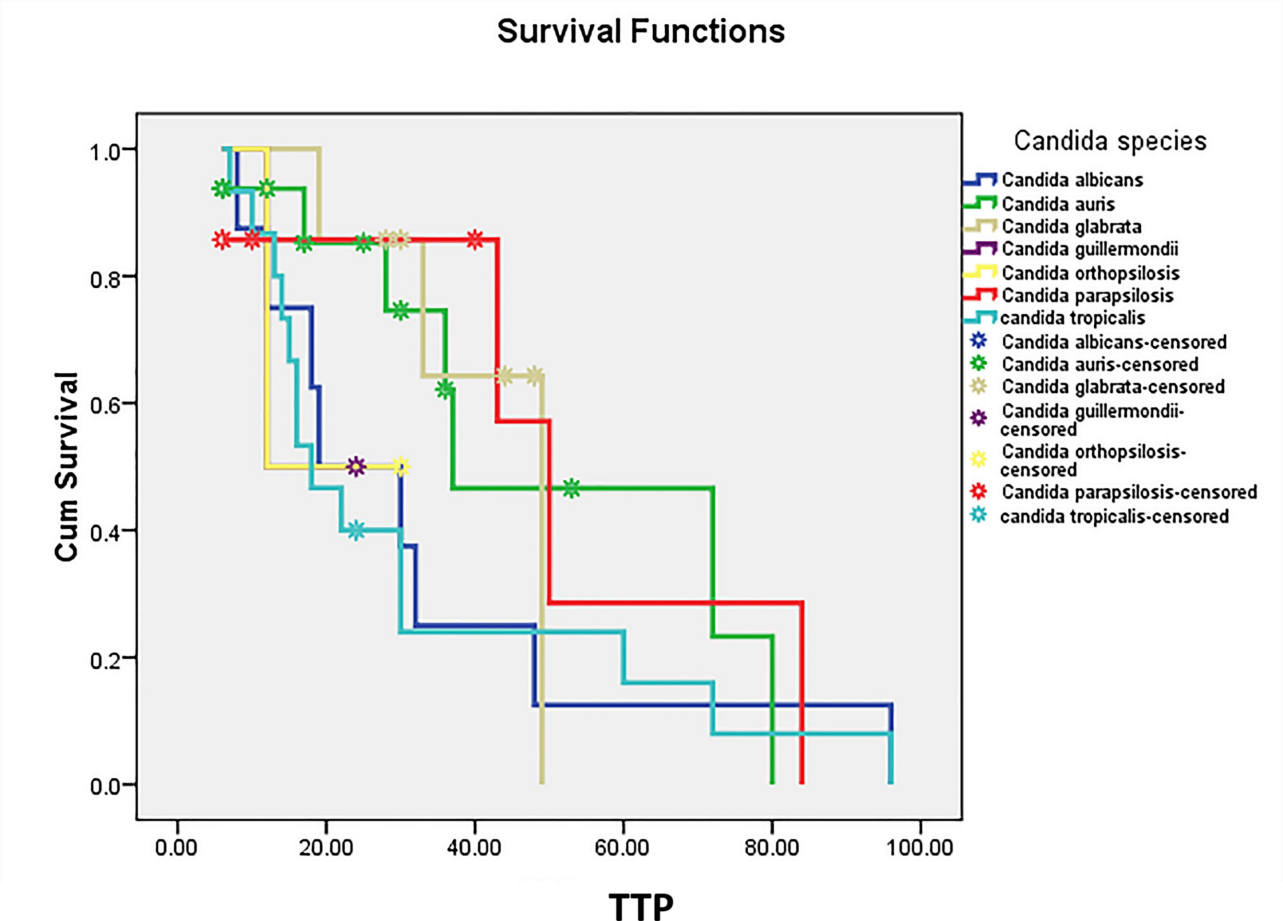
Kaplan–Meier survival curves based on the time to positivity across different Candida species. The vertical axis of a Kaplan-Meier survival curve represents the cumulative survival probability and is scaled from 0 to 1, where: 1 represents 100% survival, and 0 represents 0% survival. The horizontal axis of the Kaplan-Meier survival curve represents the time to positivity and is ranged from 0 to 100 hours (The blood culture bottles are usually incubated for 5 days, i.e., 120 hours). The curve is a step function. Drops in the curve correspond to each subject who has tested positive for* Candida*. Data points marked as "censored" indicate subjects who have died and are not included in the denominator at those time points. TTP: Time to positivity

For all episodes of candidemia, the median TTP was 24 hours, with an interquartile range of 28.5 hours. Table 5 presents 30-day mortality rates and median Time to Positivity (TTP) for different Candida species. Out of eight cases, *Candida albicans *showed no mortality (0%, 0/8), with a median TTP of 28.5 hours (IQR: 16.5-36). On the other hand, *Candida auris* had a high mortality rate of 56.25% (9/16) and a median TTP of 16.5 hours (IQR: 10.75-57.75). *Candida glabrata* also exhibited a high mortality rate of 57.1% (4/7) with a median TTP of 20 hours (IQR: 14.5-46). Similarly, *Candida guilliermondii* presented a 50% mortality rate (1/2) and a median TTP of 18 hours (IQR: 15-21), while *Candida orthopsilosis* also showed a 50% mortality rate (1/2) with a median TTP of 21 hours (IQR: 16.5-25.5). *Candida parapsilosis* showed a 42.85% mortality rate (3/7) and the shortest median TTP of 10 hours (IQR: 8-46.5). Conversely, *Candida tropicalis* had a relatively low mortality rate of 6.6% (1/15) with a median TTP of 28 hours (IQR: 17.25-37.25). Kaplan-Meier survival curves were employed to estimate the cumulative survival probabilities of all *Candida* species based on their time to positivity (Figure 1). These demonstrate that shorter TTP was associated with increased mortality with the species-dependent association. A significant p-value (0.01) from the log-rank test suggests that there is a statistically significant difference between TTP and increased mortality with the species-dependent association. *Candida auris*, which had a short time to positivity at 16.5 hours, exhibited the highest mortality rate at 56.25%. Similar findings were observed in other Candida species which have been tabulated in Table 5. Logistic regression modelling revealed that Time to Positivity (TTP) significantly influences 30-day mortality. A one-day reduction in TTP is associated with a 1.397-fold increase in the odds of mortality (95% CI: 1.08-1.741).

**Table 5:** 30-day mortality analysis of TTP with Candida species. TTP: Time to positivity IQR: Interquartile range The data is presented in (n/n) %.

Candida species	30-day mortality % (n/n)	Hours TTP Median (IQR Range)
Candida albicans	0 (0/8)	28.5 (16.5-36)
Candida auris	56.25 (9/16)	16.5 (10.75-57.75)
Candida glabrata	57.1 (4/7)	20 (14.5-46)
Candida guilliermondii	50 (1/2)	18 (15-21)
Candida orthopsilosis	50 (1/2)	21 (16.5-25.5)
Candida parapsilosis	42.85 (3/7)	10 (8-46.5)
Candida tropicalis	6.6 (1/15)	28 (17.25-37.25)

## Discussion

In our study, the isolation rate of* Candida* from blood cultures during the one-year period was 2.47% from patients suspected of having sepsis. This isolation rate is consistent with various studies conducted across different regions in the country [13-15]. Our findings revealed that candidemia was more prevalent in males (66.7%) and the 40-60 years age group (40.4%). The age and gender distribution of our study correlates with the observation of Bhattacharjee [16]. In our study, long-term antibiotic use was the most common risk factor (33%), followed by the presence of a central venous catheter (21%). These findings are consistent with studies by Xess et al. [17], Giri et al. [18] and Chowta et al. [19], which similarly highlighted the impact of prolonged antibiotic use and invasive devices on candidemia risk. The frequency of NAC isolation has been rising, correlating with higher mortality rates due to increased virulence and reduced susceptibility to antifungal medications. Our study found that *C. auris* (28%) was the most prevalent species, followed by* C. tropicalis* (26.3%), *C. albicans* (14%), *C. glabrata* (12.3%), and* C. parapsilosis* (12.3%). These findings align with research by Shastri et al. [20] and Rudramurthy et al. [21], which also reported high prevalence rates of *C. auris *and *C. tropicalis.*

The study's focus on the relationship between the Time to Positivity (TTP) of blood cultures and clinical outcomes in patients with candidemia offers valuable insights into the prognostic significance of TTP. One of the primary strengths of examining TTP is its role as a surrogate marker for microbial load and infection severity. The statistically significant association between shorter TTP and increased mortality is a crucial aspect of this study. The median TTP for deceased patients was 24 hours, compared to 25 hours for survivors, with a P-value of 0.001, indicating that patients with more severe infections tend to present with quicker positive blood cultures. These results are consistent with other studies indicating that shorter TTP correlates with more severe infections and poorer outcomes in candidemia patients [22]. This finding underscores the clinical utility of TTP as an early indicator of severe infections, enabling healthcare providers to prioritize and escalate care for high-risk patients promptly.

The study also elucidates a critical relationship between the 30-day mortality rates and the time to positivity (TTP) of blood cultures among different *Candida *species isolated from bloodstream infections. *Candida albicans* demonstrated no mortality (0%, 0/8), with a median TTP of 28.5 hours (IQR: 16.5-36), suggesting this species is associated with better clinical outcomes. In stark contrast,* Candida auris* exhibited a high mortality rate of 56.25% (9/16) and had a median TTP of 16.5 hours (IQR: 10.75-57.75), indicating a more severe infection profile. *Candida glabrata *also showed a high mortality rate of 57.1% (4/7) with a median TTP of 20 hours (IQR: 14.5-46), further highlighting its clinical severity. This trend, where species with shorter TTPs tend to have higher 30-day mortality rates, is significant. The median TTP for all isolates was 24 hours (IQR: 12-40 hours). A TTP of ≤ 24 hours is particularly associated with higher mortality, as supported by the study of Kim et al. [23].

Data regarding the TTP for *Candida* species are limited. Ben-Ami et al. [24] highlighted the utility of TTP for diagnosing catheter-related candidemia, finding that a TTP of ≤30 hours was a sensitive but non-specific indicator of an intravascular catheter source. Our study corroborates the significant difference in TTP in catheter-associated candidemia. Taur et al. [25] suggested that a longer incubation period might be associated with higher mortality, but their findings conflicted with ours and other studies on bacterial bloodstream infections [23,26].

The study acknowledges several potential confounding factors that could influence the interpretation of the relationship between Time to Positivity (TTP) and mortality in candidemia. One significant factor is the patient’s overall health and comorbidities, which can affect both the progression of candidemia and the immune response. Patients with multiple underlying health conditions might exhibit different TTPs due to varied immune responses, complicating the direct correlation between TTP and mortality. Additionally, differences in hospital practices, such as the timing of blood culture collection, volume of blood collected, transportation of the samples and the speed of laboratory processing, can impact TTP measurements. Variations in antifungal treatment protocols and patient management strategies across institutions might also influence outcomes, adding complexity to the analysis. The presence of other infections or co-infections is another confounding factor that can affect the clinical course of candidemia. Patients with concurrent bacterial or other fungal infections may have altered TTPs and mortality rates, making it challenging to isolate candidemia-specific trends. Furthermore, the use of antifungal prophylaxis in high-risk patients could reduce fungal load at the time of blood culture collection, potentially skewing TTP results. Confounding variables can be addressed by implementing standardized operating protocols in laboratories to minimize delays in sample transportation and processing. Establishing uniform treatment protocols and management strategies for bloodstream infections, along with stringent infection control practices, can also help reduce mortality rates. Research into pre-analytical factors, like sample handling and patient preparation, can help standardize conditions and improve TTP measurement reliability. Integrating TTP data with clinical variables, such as comorbidities and treatment regimens, could enhance its prognostic value. Investigating advanced diagnostic techniques, such as the T_2_ *Candida* magnetic resonance assay, could enhance the speed and accuracy of *Candida* identification. This assay can rapidly detect the five most commonly isolated* Candida *species directly from whole blood in approximately 5 hours, potentially leading to faster diagnoses and improved early treatment outcomes.

Despite these confounding factors, TTP remains a critical surrogate marker of microbial load and infection severity. Our data strongly indicate that shorter TTP is associated with higher 30-day mortality rates cumulatively across different* Candida *species. This relationship underscores the potential of TTP as a prognostic marker in candidemia. Early detection of candidemia (short TTP) often correlates with severe infections and worse outcomes, emphasizing the need for prompt and aggressive treatment, especially for species like* C. auris, C. parapsilosis *and* C. glabrata.*

Limitations of the study

The study on Time to Positivity (TTP) and mortality in candidemia has several limitations. Firstly, the small sample size limits the statistical power, making it more challenging to detect significant associations between TTP and 30-day mortality. Consequently, the findings may not be generalizable to broader populations or other healthcare settings, as the results are more susceptible to variability and bias. Additionally, subgroup analyses are difficult to perform due to the limited number of cases, leading to less precise estimates with wider confidence intervals. Limited coverage of antifungal susceptibility profiles restricts the generalizability of the findings. Data from a single medical centre may not represent other regions or healthcare settings, impacting the results. These limitations must be considered when interpreting the study’s results and their implications for clinical practice.

## Conclusions

In conclusion, our analysis supports the conclusion that a shorter TTP is associated with increased mortality in candidemia, with species-specific differences influencing TTP. The examination of TTP in relation to mortality in candidemia patients provides a valuable prognostic indicator, although it must be interpreted within the context of potential confounding factors. The study's strengths lie in its detailed analysis of species-specific data and its alignment with existing research, reinforcing the clinical relevance of TTP. By addressing these variables and acknowledging the limitations, the study offers a comprehensive perspective on the utility of TTP in guiding the management of candidemia, highlighting its potential to enhance patient care through timely and targeted interventions.
